# Mechanisms Underlying the Expansion and Functional Maturation of β-Cells in Newborns: Impact of the Nutritional Environment

**DOI:** 10.3390/ijms23042096

**Published:** 2022-02-14

**Authors:** Cécile Jacovetti, Romano Regazzi

**Affiliations:** 1 Department of Fundamental Neurosciences, University of Lausanne, 1005 Lausanne, Switzerland; cecile.jacovetti@unil.ch; 2 Department of Biomedical Sciences, University of Lausanne, 1005 Lausanne, Switzerland

**Keywords:** postnatal β-cell development, β-cell proliferation, β-cell maturation, in utero and postnatal obese environment, embryonic nutritional deficiency, transgenerational metabolic disorders

## Abstract

The functional maturation of insulin-secreting β-cells is initiated before birth and is completed in early postnatal life. This process has a critical impact on the acquisition of an adequate functional β-cell mass and on the capacity to meet and adapt to insulin needs later in life. Many cellular pathways playing a role in postnatal β-cell development have already been identified. However, single-cell transcriptomic and proteomic analyses continue to reveal new players contributing to the acquisition of β-cell identity. In this review, we provide an updated picture of the mechanisms governing postnatal β-cell mass expansion and the transition of insulin-secreting cells from an immature to a mature state. We then highlight the contribution of the environment to β-cell maturation and discuss the adverse impact of an in utero and neonatal environment characterized by calorie and fat overload or by protein deficiency and undernutrition. Inappropriate nutrition early in life constitutes a risk factor for developing diabetes in adulthood and can affect the β-cells of the offspring over two generations. A better understanding of these events occurring in the neonatal period will help developing better strategies to produce functional β-cells and to design novel therapeutic approaches for the prevention and treatment of diabetes.

## 1. Introduction

The acquisition of a suitable and fully functional adult pancreatic β-cell mass is a fundamental prerequisite for the maintenance of carbohydrate homeostasis and for facing the pathophysiological challenges encountered by our organism throughout life [1,2]. The postnatal period is a key window where the endocrine pancreas is expanding to reach an appropriate adult β-cell mass and in which insulin-secreting cells finalize their functional maturation [3,4]. These processes of proliferation and functional maturation are vital to cover the insulin requirements in adulthood in adequacy with the transition from infant to adult nutrition. Furthermore, these processes are essential in response to critical changes in body metabolism occurring during pregnancy or following the appearance of metabolic disorders including diabetes, obesity, metabolic syndrome, other endocrine disorders, or even aging. A deleterious nutritional environment during fetal and postnatal life has harmful repercussions that can predispose to the development of diabetes during childhood and adulthood, a predisposition that can even be transmitted to the next generation [5].

## 2. Literature Search Strategy

We conducted our bibliographic research exclusively via PubMed using various keywords such as « postnatal β-cell development », « β-cell development », « postnatal β-cell mass », « β-cell maturation », « nutritional switch during weaning », « diabetes predisposition », « fetal environment and diabetes risk », « maternal obesity », « maternal undernutrition », and « transgenerational inheritance of diabetes ». We also analyzed papers based on the bibliographic references cited by the studies found on PubMed during our search. Whenever possible, we selected the most recent and comprehensive reviews on the topic in question. All selected articles were written in English.

## 3. Insights into the Intracellular Mechanisms Driving Early Postnatal β-Cell Mass Expansion and Maturation

**Figure 1:**
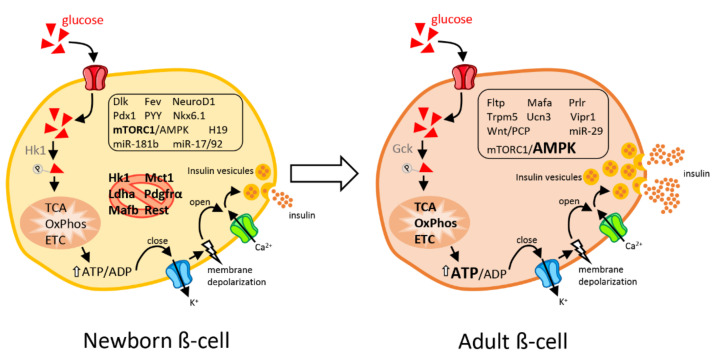
Schematic view of an immature β-cell from newborns and a mature, fully functional β-cell from adults. Newborn β-cells display elevated replicative capacities but are poorly efficient in secreting insulin in response to a rise in blood glucose levels. Disallowed genes (crossed-out by a red circle) are repressed in immature neonatal β-cells to permit β-cell maturation. In adults, β-cells are fully competent in releasing insulin in response to glucose but have a very limited proliferative capacity. In response to elevated glucose concentrations, β-cells metabolize glucose through glycolysis, TCA (tricarboxylic acid cycle), OxPhos (oxidative phosphorylation), and mitochondrial ETC (electron transport chain) which leads to an increased ATP/ADP ratio. The rise in cytosolic ATP results in closure of ATP-sensitive potassium channels, membrane depolarization and opening of voltage-gated Ca^2+^ channels. The resulting calcium influx triggers insulin-containing dense core granule exocytosis. The main transcription factors, signaling pathways and non-coding RNAs responsible for the phenotype of immature versus mature β-cells are shown in the figure. Genes and non-coding RNAs that are enriched in each cell type are framed. A comprehensive list of the gene abbreviations is provided at the end of the manuscript. Mirror changes between neonatal and adult β-cells are highlighted in bold.

In mammals, expansion of the endocrine pancreas and β-cell maturation are initiated at the embryonic stage but continue during early postnatal life to achieve the functional mass of insulin-secreting cells required in adults. The physiological and histological changes associated with the events occurring in the postnatal period have already been extensively discussed [1]. In the current review, we will primarily focus on the molecular mechanisms and cellular signaling pathways underlying these phenotypic changes (Figure 1). It has been reported that the transcriptomic profile of islets in neonates differs significantly from that of adult islets in both rodents and humans [6,7,8,9,10,11]. In particular, a considerable amount of transcriptomic data points to an inverse correlation between immature cells that are highly proliferative with a low ability to secrete insulin in response to elevated glucose concentrations versus mature adult cells demonstrating low proliferative adaptation capacities, but which are fully functional [11,12].

### 3.1. β-Cell Heterogeneity during Postnatal Development and “Immaturity Signature”

New insights into the notion of β-cell heterogeneity during postnatal development have emerged thanks to the possibility to perform single-cell RNA sequencing from primary tissues [3]. Single-cell sequencing of fluorescence-activated cell sorting (FACS)-sorted β- and α-cells from mice at different stages of development including embryonic day 17.5 (E17.5), postnatal day 0 (P0), P3, P9, P15, P18, and P60 revealed heterogeneity in gene expression allowing to categorize the cells into two main groups [13]. At P0, P3, and P9, a first group of β-cells show a high abundance of genes associated with an immature phenotype such as *Mafb* (basic leucine zipper (bZIP) transcription factor B), *Pyy* (peptide YY), and *Fev* (fifth ewing variant) while a second group of cells express predominantly genes such as *Trpm5* (transient receptor potential cation channel subfamily M member 5), *Vipr1* (vasoactive intestinal polypeptide receptor 1), and *Prlr* (prolactin receptor), known to be abundant in so-called mature and fully operational cells. Despite this identified transcriptomic heterogeneity, neonatal β-cells appear to follow a synchronous functional maturation process. Gene ontology analysis at the bulk-cell level confirms the dichotomy between neonatal and adult cells with an enrichment of genes involved in cell migration, cell adhesion, and cell differentiation pathways in newborn cells and predominant expression of genes associated with metabolic processes, transport and hormone secretion in 60-day old mouse β-cells [13]. 

In addition to the activation of transcriptional regulatory mechanisms during postnatal maturation, there is emerging evidence indicating that post-transcriptional events may also contribute to the acquisition of the mature β-cell phenotype. *Fltp* (also known as Flattop and *Cfap126*), a wingless/integrated/planar cell polarity (WNT/PCP) downstream factor, has been established as a marker to distinguish between immature/proliferative (Fltp-) and mature/quiescent (Fltp+) cells [14]. At postnatal day 1, the PCP pathway is weakly active in mouse β-cells while a strong activity is observed in adults, which is inversely correlated to the proliferation capacity [14]. However, no major transcriptomic differences between cells expressing low or high levels of *Fltp* could be detected [13]. Therefore, it is likely that the WNT/PCP pathway drives the establishment of planar polarization and the acquisition of a mature cell phenotype by regulating post-transcriptional rather than transcriptional events [15].

### 3.2. New Insights into the Molecular Mechanisms of β-Cell Replication in Newborns

Many factors have been identified as key regulators of islet cell proliferation. The signaling pathways triggered by these factors are particularly active during postnatal β-cell development and decline with age [11,13,16]. A signaling pathway elicited by Glucagon-like peptide 1 (GLP1) is specifically activated in juvenile β-cells in which exendin-4, a GLP1 analogue, induces the expression of genes involved in proliferation. Adult β-cells treated with exendin-4 do not activate this molecular cascade, which may contribute to their restricted proliferative capacity [17]. In vitro and in vivo experiments show that ectopic expression of the transcription factor *c-Myc* (myelocytomatosis viral proto-oncogene, BHLH transcription factor) induces an increase in the level of genes involved in cell cycle control, coding for components of the biosynthetic machinery and of proliferation markers, thereby reactivating the replication capacity of adult β-cells [12]. In these cells, maturation markers that are highly expressed during postnatal cell development are induced (pancreatic and duodenal homeobox 1 (Pdx1), NK6 homeobox 1 (*Nkx6.1*), neuronal differentiation 1 (*Neurod1*), basic leucine zipper (bZIP) transcription factor A (*Mafa*) and urocortin 3 (*Ucn3*)) while the level of genes vital for the maintenance of the secretory activity is reduced. *C-Myc* over-expressing cells have a low capacity for insulin secretion but proliferate three times more than control cells [12]. Reversing the quiescent proliferative state of mature cells is also possible via ectopic expression of the long non-coding RNA H19, which is abundant in newborn rat islet cells and is “silenced” in adult β-cells [18]. Overexpression of H19 promotes the proliferation of adult β-cells, most likely by sequestering the microRNA let-7 and increasing serine/threonine protein kinase (AKT) phosphorylation, two important regulatory events in β-cell replication [18,19]. A signaling pathway involving the mitogen activated protein 3 kinase 12, also called dual leucine zipper-bearing kinase (DLK), has been recently identified as a regulator of postnatal β-cell proliferation. Several studies observed a very high expression and activity of DLK in the cytoplasm of human and rodent neonatal β-cells [10,20,21]. DLK leads to the activation of c-Jun N-terminal kinase 3 (JNK3) and an increase in the expression of the cyclins (cyclin D1) *Ccnd1* and (cyclin D1) *Ccnd2*, a signaling pathway essential in β-cell replication in the postnatal period. The repression of DLK in islets of neonatal rats reduces the capacity of β-cells to proliferate in the postnatal period [21]. Furthermore, the levels of *DLK*, *JNK3*, *CCND1* and *CCND2* are higher in the islets of obese non-diabetic patients undergoing compensatory β-cell mass expansion versus thin or obese diabetic individuals [21,22,23]. Several studies showed that the gene expression profile of β-cells from patients suffering from metabolic disorders displays many transcriptomic similarities with immature neonatal β-cells [8,20,21].

### 3.3. Signaling Pathways Driving the Acquisition of Functional β-Cell Features

Recent work carried out on human material allowed to monitor the transcriptomic profile of islets in neonates, adolescents, and adults [8]. The major transcriptomic changes observed during the maturation process strongly support the functional immaturity of neonatal islets. These include low expression of genes coding for mitochondrial shuttles (malate dehydrogenase, glycerol-3-phosphate dehydrogenase, glutamate oxaloacetate transaminase, malate-aspartate-NADH (nicotinamide adenine dinucleotide)) [8,10,24], for key enzymes involved in the metabolism of glucose (pyruvate carboxylase, glucose-6 phosphatase 2), and fatty acids (carnitine palmitoyl transferase 2, fatty acid-binding protein 5 (*FABP5*)) [8,24], for components of the calcium signaling pathway [25], for transcription factors [26] such as *MAFA* and *PDX1* involved in insulin biosynthesis and secretion [27], as well as for different classes of non-coding RNAs including PIWI-interacting RNAs, microRNAs, and long non-coding RNAs [18,28,29]. Extensive characterization of the transcriptomic profile coupled with the analysis of histone marks in the promoters of genes associated with endocrine cell maturation from human juvenile and adult pancreas confirmed age-specific changes in the expression of β-cell transcription factors, including *MAFA* [20]. This study also identified factors specific to human β-cells, not expressed in mouse islet cells, which increase during postnatal maturation. Overexpression of one of them, the transcription factor Sine Oculis homeobox homolog 3 (SIX3), in juvenile β-cells increased the insulin secretory capacity whereas loss of SIX3 led to the induction of genes associated with an immature β-cell state that is most commonly found in non β-cells [30]. Altogether, this work provides valuable information on novel regulators specifically driving the functional maturation of human β-cells and validates the findings from numerous rodent studies. Due to the scarcity of juvenile human material and the much longer duration of the process of β-cell maturation in humans [8,20,30], it was important to verify the reliability of rodent models. In fact, in rodents, postnatal changes occur over a much shorter period and weaning is a much more drastic event compared to the progressive dietary diversification occurring in humans.

The laboratories of Claes Wollheim, Guy Rutter, Frédéric Lemaire and Franz Schuit, among others, made major contributions in identifying a pool of so-called “disallowed genes” that need to be silenced to achieve a fully functional β-cell. Several of these transcripts are expressed at a high level in β-cells of newborn rodents and humans [8,31]. Elevated levels of the lactate dehydrogenase (LDHA) and the monocarboxylate transporter 1 (MCT-1) specifically affect glucose-induced insulin secretion [31]. Indeed, in β-cells, the increase of these proteins interferes in the coupling of the glycolytic flow with mitochondrial oxidative phosphorylation [32,33]. In β-cells, the expression of MCT-1 allows pyruvate and lactate produced by muscles during exercise to trigger oxidative phosphorylation, resulting in an inappropriate rise in insulin secretion [34]. The gene coding for MCT-1, solute carrier family 16 member 1 (*SLC16A1*), is strongly repressed in adult β-cells of healthy subjects and individuals with high levels of MCT-1 suffer from exercise-induced hypoglycemia [35]. Neonatal β-cells do not show a predominant glycolytic flux on oxidative metabolism (Crabtree effect) [36], nor a glycolytic overproduction of lactate in anaerobic conditions (Pasteur effect) [37]. The silencing of genes such as *Ldha* and Hexokinase 1 (*Hk1*) during β-cell maturation is driven by the methylation pattern of their loci controlled by the DNA methyltransferase DNMT3A [38]. The microRNA miR-29 appears also to play an important role in the control of postnatal β-cell maturation by repressing different disallowed genes and by inhibiting, among others, the expression of *Mct-1*, *Pdgfra* (platelet derived growth factor receptor alpha), and *Rest* (RE1 silencing transcription factor) [6,29,39,40]. The level of this microRNA is higher in rat islets after weaning. Indeed, the expression of miR-29 and of several other microRNAs seems to be strongly affected by the switch to a carbohydrate-rich diet induced by weaning [29]. 

Beside miR-29, several other microRNAs contribute to the epigenetic mechanisms operating during the functional transition of β-cells allowing the adaptation to the changes in nutritional intake and in insulin requirements associated with weaning [29,41]. The expression profile of numerous key transcription factors also depends on the methylation status of the genome and the subsequent stability of the transcriptome. The activity of histone methyltransferases such as RNA methyltransferase-like 3/14 (METTL3/14) and Histone H3 lysine K4 (H3K4) has been shown to be essential both for terminal differentiation and expansion of β-cells in the postnatal period via the regulation of the expression of *Mafa* [42,43]. 

### 3.4. Environmental Drivers of the Transition from Immature to Mature Postnatal β-Cell Function

The transition from a lipid-dominant to a carbohydrate-rich diet in the postnatal period appears as a crucial determinant in the transcriptomic changes associated with the functional maturation of adult β-cells. Weaning leads to an enrichment of genes coding for mitochondrial electron transport chain proteins and genes involved in the transition from replication to quiescence (e.g., *Mcm3/4/6/7/10* (minichromosome maintenance complex component), *Prim1* (DNA Primase Subunit 1), *Cdt1* (chromatin licensing and DNA replication factor 1), *Orc5*, and *Orc6* (origin recognition complex subunit 5/6)) [11]. These changes are consistent with the fine tuning of insulin secretion in response to high glucose and with the acquisition of a glucose-dependent replication capacity of β-cells observed after weaning [11]. Nonetheless, these transcriptomic changes leading to the acquisition of β-cell function appear not to be directly linked to the level of well-established markers of β-cell differentiation such as *Mafa* [27], *NeuroD1* [44], and *Ucn3* [45]. This is also the finding of a study in which the authors focused on the ability of immature β-cells of mouse and human neonates to secrete insulin in response to amino acids but not to glucose. This amino acid-dependent effect is achieved by modulating the activity of rapamycin complex 1 (mTORC1), which fluctuates in response to changes in the nutritional environment [38,46]. Inactivation of mTORC1 following the nutritional transition occurring at weaning favors the activation of 5′-Adenosine monophosphate-activated protein kinase (AMPK) [38], an inhibitor of mTORC1 activity and a sensor of the cellular energy status [47,48]. The switch in the mTORC1/AMPK ratio is responsible for the gradual transition of the secretory capacity of immature β-cells to a mature insulin secretion profile. Indeed, as a result of these molecular changes, immature β-cells that release insulin primarily in response to amino acids acquire the capacity for glucose-dependent insulin release, a property unique to adult and fully mature β-cells [38,46,48]. Again, the specificity of the glucose response is not directly associated with the levels of the differentiation markers PDX1, NKX6-1, UCN3, and MAFA that are not altered under conditions promoting the functional maturation [46]. 

In addition to cell-autonomous mechanisms, the islet niche appears to be a source of circulating factors which contribute to the acquisition of β-cell-specific functions. Pericytes that line the endothelium of blood capillaries produce the Bone Morphogenetic protein 4 (BMP4) and activate a downstream signaling pathway in β-cells that boosts the expression of key metabolic genes such as *Mafa*, *Pdx1*, *NeuroD1*, and *Nkx6.1* [49]. Indeed, BMP4 increases the ability of β-cells to secrete insulin in a transgenic mouse model and in human induced pluripotent stem cells (iPSC)-derived β-like cells [49]. Fetuin-A is a glycoprotein mainly secreted by the liver during fetal life, and whose serum concentration declines in the postnatal period. Recently, this protein was identified as an inhibitor of glucose-induced insulin secretion and β-cell proliferation via a decrease of TGFBR (transforming growth factor beta receptor)-SMAD2/3 (TGFB signaling protein, mothers against decapentaplegic homolog 2/3) signaling and transcriptomic repression of maturity markers (*NeuroD1*, *Ucn3*, *Abcc8* (ATP binding cassette subfamily C member 8) and *Casr* (calcium-sensing receptor)) and regulators of cell replication (*Foxm1* (forkhead box protein M1), *Cenpa* (centromere protein A), *Cdk1* (cyclin-dependent kinase 1) or *Top2a* (DNA Topoisomerase II Alpha)) [50]. Thus, fetuin-A is potentially a circulating factor involved in the transition to functionally mature β-cells. Thyroid hormone (TH) has a similar potential for coordinating α- and β-cell maturation during the larval-to-juvenile transition in zebrafish, pointing to the preservation among species of the role of TH in the development and maintenance of carbohydrate homeostasis [51]. Altogether, these recent studies further highlight the importance of remodeling that pancreatic β-cells undergo under the control of exogenous factors [49]. 

Nutrients do also play an important regulatory role. In utero environment and maternal milk are rich sources of amino acids and lipids. The expression of microRNAs in neonatal β-cells can also be regulated by the nutritional environment in the postnatal period with the modulation of microRNAs such as the members of the miR-17/92 cluster, miR-194-5p, miR-181b-5p, and miR-129-5p. These microRNAs have been shown to play a role in the establishment of insulin secretion and in the replication capacity of immature β-cells [29]. Premature exposure of pups to a carbohydrate-rich diet alters the levels of these microRNAs and induces early insulin secretion capacity. Conversely, prolongation of a diet rich in lipids of nutritional composition close to breast milk delays the transcriptomic changes and the functional maturation of β-cells [29]. 

Thus, after birth, β-cells show a great adaptation to nutritional challenges that occur in parallel with the intrinsic maturation process driven by a well-established transcriptional reprogramming that we have outlined above [41,46]. In addition, certain environments during fetal and neonatal life induce transcriptomic changes within the maturing islets, which will potentially influence the ability of β-cells to adapt to different environmental stimuli in adulthood and thus promote the susceptibility of individuals to develop metabolic disorders. 

## 4. Inappropriate Fetal and Postnatal Environment Induces Persistent Changes That Increase Type 2 Diabetes Risk

In utero environment strongly influences the multiple signaling pathways required for embryonic and postnatal development of the endocrine pancreas that have been described above. The quality and adequacy of intrauterine conditions are determined in large part by maternal metabolic health status and nutrition. Thus, diabetes, obesity, and over- or undernutrition during pregnancy may be responsible for many alterations in pancreatic development and significantly increase the susceptibility of the offspring to develop metabolic diseases in adulthood such as metabolic syndrome, obesity, cardiovascular disease, and Type 2 diabetes (T2DM). The incidence of overweight in the Western world with eating habits based on a Western Diet which favors weight gain in association with inadequate physical activity is reaching alarming levels. The molecular mechanisms that lead to functional defects in pancreatic endocrine cells of newborns exposed to excess or insufficient nutrients throughout embryonic and postnatal life are not yet fully understood. However, numerous studies in human cohorts (Table 1) but also in non-human primates [52], rodents [5,53,54], as well as in in silico models [55] have refined our understanding of the genetic, epigenetic, cellular, and physiological mechanisms involved in this “transgenerational diabetic programming”. Indeed, chronic exposure to a hyperglycemic or nutrient-deficient intrauterine environment determines the physiological response of the offspring and advocates the risk of developing metabolic diseases in adulthood, a predisposition that may even carry over to future generations. This concept of “in utero fetal programming” is often referred to as the “thrifty phenotype” or “metabolic memory” [56]. In the following sections, we will discuss the molecular and cellular mechanisms that are potentially underlying this metabolic memory.

### 4.1. In Utero and Postnatal “Obesogenic” Environment has a Deleterious Impact on β-Cells of the Progeny

**Figure 2:**
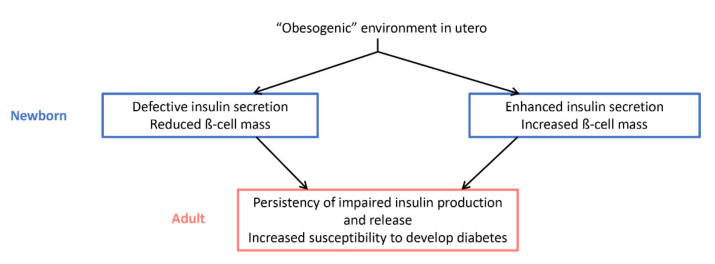
Impact of an “obesogenic” environment in utero on glucose homeostasis and diabetes susceptibility at birth and in adulthood. Depending on the species and whether the phenotype of the animals was studied distinguishing males from females, the effects observed at birth diverge. However, there is a consensus regarding the alteration of insulin physiology in adulthood and the susceptibility to diabetes.

Transcriptional and physiological alterations are already present at birth following exposure to an “obese in utero environment”. Although results differ between studies as to whether the β-cell mass of newborns exposed to an “obesogenic” intrauterine environment is increased or reduced, there is a consensus that β-cell function is affected (Figure 2) [54,77,78]. Billestrup’s group observed an increased expression of pro-inflammatory factors including phospholipase A2 (*Pla2*) and of the interleukin 1 receptor antagonist (*Il-1ra*) in the pancreas of the offspring of mice fed with a high-fat diet, which may impair pancreatic endocrine function [79]. Nevertheless, most studies that have investigated the consequences of inappropriate fetal and postnatal environment on the offspring of mothers with obesity or malnutrition have focused on the persistency of the phenotype in adulthood and the susceptibility to develop T2DM later in life. A non-human primate model with human-like architectural features was also used to characterize the functional alterations occurring upon exposure to an “obesogenic” maternal environment [80]. The authors of this study reported a decrease in α-cell proliferation and mass in female Japanese Macaques (Macaca fuscata) of 3 years of age after exposure to a Western-Style Diet in utero and during lactation [52]. However, in this model, glucose homeostasis of males and females is less drastically altered than in rodents since, despite higher glucose excursions, the animals are not glucose intolerant. An important point is the ex vivo finding that the islets of the offspring of non-human primates exposed to hyperlipidic maternal over-nutrition secrete more insulin than the islets of the offspring of control mothers, a phenotype that may be related to the reduction of the α-cell mass secreting glucagon, a hormone with antagonistic effects to insulin. These results align very well with the recently published observation made in F1 female mice born from mothers fed an obesogenic diet prior to and throughout pregnancy and lactation [81]. However, important sex differences appear to be present in rodents. Indeed, in agreement with the observations made in macaques, the males in the offspring also had a reduced α-cell mass with no significant glucose intolerance. However, they showed decreased expression of L-type Ca^2+^ channels (*Cacna1c* and *Cacna1d* (calcium voltage-gated channel subunit alpha1 C/D)), less insulin granules docked at the plasma membrane, as well as defective mitochondrial respiration associated with decreased ATP production [81]. On the contrary, the offspring adult females displayed an increase in insulin secretion in response to glucose and leucine/glutamate amino acids, elevated mitochondrial respiration with increased expression of electron transport chain components and antioxidant enzymes, and expressed more estrogen receptors and reduced markers of apoptosis [81]. In contrast to the data in primates [52], several studies in rodents reported upon maternal high-fat diet (HFD, equivalent to Western-Style Diet) an improved insulin secretory capacity in both sexes, with increased insulin secretion in adulthood in F1 and F2 generations [78,82] which was associated with an increase in β-cell mass [82,83,84]. However, the consensual observation of a decline over time in adult β-cell function with the onset of glucose intolerance [77], which is linked to the susceptibility to develop diabetes, appears to reflect the loss in β-cell function reported in T2DM patients [85]. A characterization of the signaling pathways that are affected at adulthood in the islets of the offspring confirms the alterations already observed in the neonatal period and in the islets of diabetic patients. Indeed, in the islets of the offspring of obese mothers, there is a persistent reduction in the expression and/or activity of key factors such as *Pdx1* and *NeuroD1* [82,86], the enzymes glyceraldehyde-3-phosphate dehydrogenase (*Gapdh*) and transketolase (*Tk*), involved in glucose metabolism [87], markers of inflammation (interleukin 1 beta (*IL1beta*), C-C Motif Chemokine Ligand 2 (*Ccl2*)), mitochondrial function and resistance to oxidative stress (ATP synthase peripheral stalk-membrane subunit B (*Atp5f1*), superoxide dismutase 2 (*Sod2*)), and of ribosomal proteins (*Rps6*, *Rps14* (ribosomal protein S6/14)) [88]. The increased risk of perturbed glucose homeostasis persists up to the F2 generation [82,86]. 

Father obesity has also been shown to be a risk factor for the development of metabolic disorders in the following generation [89]. Female Sprague Dawley neonates whose fathers were fed an HFD diet have impaired insulin secretion and glucose intolerance at adulthood. A transcriptomic analysis coupled with the analysis of the DNA methylation pattern of pancreatic islets in female offspring revealed an altered epigenome with reduced methylation of interleukin 13 receptor subunit alpha 2 (*Il13ra2*), a gene involved in key molecular pathways for pancreatic islet functions such as the JAK (janus kinase)-STAT (signal transducer and activator of transcription) signaling pathway [89,90]. This was associated with numerous changes in the expression of factors essential for glucose metabolism, apoptosis, and cell cycle as well as PI3K (phosphoinositide 3-kinase)-mTOR-signaling [89,91]. Later on, it was discovered that the consumption of an HFD diet remodels the epigenome of spermatozoa via differential DNA methylation and changes in the small non-coding RNA profile in male F0 rats that persist in the spermatozoa of F1 males [92]. 

These molecular events affect the metabolic tissues of F1 and F2 generations that show altered let-7c levels, a microRNA regulating glucose homeostasis by affecting muscle, adipose tissue, and liver function. Already at birth, F1 and F2 newborns of obese fathers display a reduced β-cell mass that lead to glucose intolerance in adulthood. Moreover, the alteration of the metabolic phenotype is exacerbated when F2 females are also fed an HFD [92].

The generational transmission of a metabolic phenotype via epigenetic modifications in sperm also involves the regulation of additional non-coding RNAs. Under certain conditions, transfer RNAs (tRNAs), which serve as amino acid carriers during protein synthesis, can be cut by endonucleases to generate tRNA fragments (tRFs) with regulatory activities [93]. The role and mode of action of tRFs in β-cells remain to be elucidated. Nevertheless, a pioneering study established a crosstalk between tRFs dysregulation in the father sperm and diabetes risk in the progeny. The expression profile of tRFs is altered in the sperm of male mice fed an HFD for 6 months beginning at 5 weeks of age leading to the development of obesity [94]. Total tRFs were isolated from the sperm and injected into control zygotes. In adulthood, animals injected at the zygotic stage with the small RNAs from obese fathers were found to be glucose intolerant and insulin resistant. Computational analysis revealed that tRFs modified in the sperm of obese males can potentially regulate 62 genes whose expression is altered in eight-cell embryos injected with RNAs isolated from the HFD F0 group, including *Maea* (macrophage erythroblast attacher, E3 ubiquitin ligase), *Ccnc* (cyclin C), and *Deptor* (DEP domain containing MTOR interacting protein), which are known regulators of β-cell function and/or associated with diabetes [95,96].

This phenomenon of transgenerational metabolic alterations associated with a process of “metabolic memory” of various cell types seems to reduce the capacity of the animals to adapt and compensate for environmental challenges during adult life (over-nutrition, pregnancy, senescence, etc.), leading to failure in the ability to maintain glucose homeostasis. Adult female mice exposed to an obesogenic but normoglycemic intrauterine environment and fed an HFD in adulthood from the 15th week of age develop glucose intolerance at 50 weeks of age, which is associated with a decrease in the amount of insulin secreted [87]. Aggravation of the metabolic phenotype caused by environmental context such as HFD has also been observed in the adult offspring of mothers with gestational diabetes [97]. The sex of the animals was not specified in this study, so we don’t know whether this was a sex-specific effect [88]. Sex differences were reported by another study which observed an inability to adapt to a high-sucrose diet leading to glucose intolerance specifically in adult male rats whose dams had been exposed to HFD during gestation [98]. 

Despite some sex and species differences, the literature points to a lack in the adaptive capacity of β-cells when the animals are subjected to over-nutrition in utero which becomes critical at adulthood in the case of environmental context such as obesity.

### 4.2. Effects of Maternal Undernutrition on β-Cells of the Progeny

**Figure 3:**
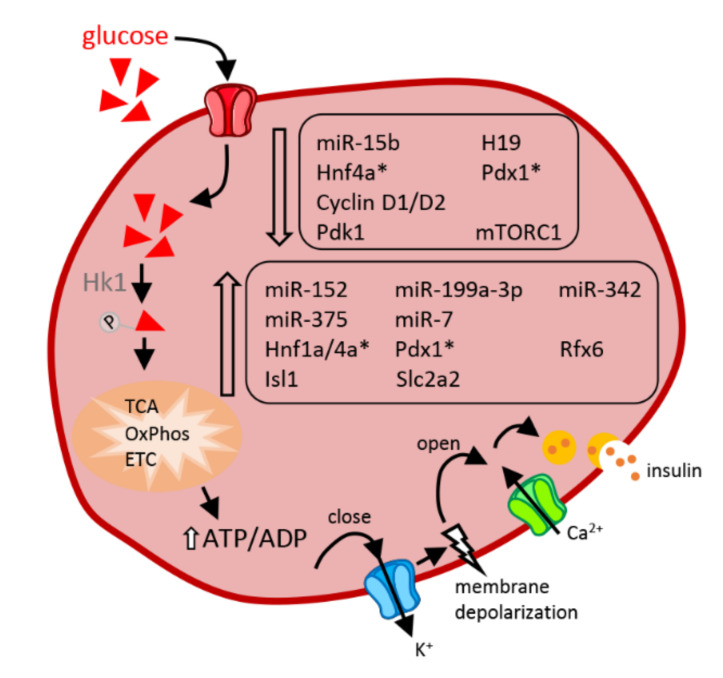
Impact of protein deficiency in utero on newborn β-cells. Non-coding RNAs, transcription factors, or key components which display enhanced (bottom frame) or reduced (top frame) expression under conditions of in utero protein deficiency are indicated in the figure. Transcription factors for which conflicting results have been reported (see details in the main text) are marked with an asterisk (*). The arrows indicate the chronology of the successive steps through which glucose leads to insulin exocytosis. Abbreviations in alphabetical order: ADP: adenosine diphosphate, ATP: adenosine triphosphate, Ca^2+^: calcium ions, ETC: electron transport chain, H19: H19 imprinted maternally expressed transcript, Hk: hexokinase 1, Hnf1a/4a: hepatocyte nuclear factor-1/4 alpha, Isl1: insulin gene enhancer protein, K^+^: a positively charged potassium ion, mTORC1: mammalian target of rapamycin complex 1, OxPhos: oxidative phosphorylation, Pdk1: phosphoinositide-dependent kinase-1, Pdx1: pancreatic and duodenal homeobox 1, Rfx6: regulatory factor X6, Slc2a2: solute carrier family 2 member 2, TCA: tricarboxylic acid cycle.

Analysis of the metabolic phenotype of children born during the famine periods of the 20th century revealed that individuals exposed to undernutrition during fetal life have a higher risk of developing metabolic diseases in adulthood, including T2DM [99]. Children born from mothers with energy deficiency during pregnancy have reduced birth weight, impaired insulin secretion at adulthood, and a predisposition to develop glucose intolerance. These disorders are accentuated when these same individuals develop obesity [99]. Murine models have been used to decipher the mechanisms underlying these metabolic disturbances (Figure 3). 

These models consist mainly in inducing maternal undernutrition by providing a restricted protein intake during gestation and lactation, or by partial occlusion of the uterine arteries known as the intrauterine growth retardation model (IUGR) [100]. Animals from mothers fed a low protein (LP) diet have reduced β-cell mass associated with decreased β-cell proliferation and are glucose intolerant at adulthood. Ex vivo their islets secrete less insulin in response to elevated glucose concentrations [101]. Differential expression of various factors, including a decrease in the expression of mitochondrial electron transport chain subunits, the transcription factor *Tfam* (transcription factor A, mitochondrial), which regulates mitochondrial DNA replication, antioxidant enzymes (glutathione and peroxiredoxin) [102], pro-proliferative growth arrest specific protein 6 (*Gas6*) [103], growth hormone receptor and insulin-like growth factor 2 [79,104], and amino acid metabolism [105], was observed in pancreatic islets of newborn rodents exposed to maternal undernutrition during fetal life. These transcriptomic alterations are in line with the defective mitochondrial activities observed in neonatal rodents subjected to fetal malnutrition either by placental artery ligation, caloric restriction, or a protein-depleted diet of the mother [79]. Just as transcripts of protein-coding genes are altered, the expression of non-coding RNAs is also sensitive to protein deficiency and appears to play a role in the loss of β-cell function in the postnatal period. This is the case for miR-15b, which has been reported to display changes in the islets of LP neonatal rats. miR-15b over-expression inhibits glucose-stimulated insulin secretion (GSIS) as well as MIN6 cell replication via direct repression of cyclin D1 and D2. Inhibition of miR-15b fully restores the secretory and proliferative capacities of LP islet cells in vitro [101]. A global analysis of microRNA expression profiling revealed an impact of the exposure to maternal undernutrition on the level of 47 microRNAs in fetal pancreatic islets of LP rats at E21 [106] and 14 microRNAs in islets of LP mice of 2 to 3 months of age [107]. Another microRNA that appears to contribute to the phenotype of LP neonates is miR-375 [106], whose function on islet cell proliferation and insulin secretion has been extensively studied [7]. MiR-375 is increased in fetal E21 islets from LP animals and is maintained elevated if the islets are kept in culture for 7 days. Interestingly, overexpression of this microRNA in adult islets suppresses their proliferation and secretory capacity, thus “mimicking” the phenotype observed in LP rats. The action of miR-375 is most likely mediated by inhibition of phosphoinositide-dependent kinase-1 (PDK-1), a direct target of this microRNA [108], and a key component of the insulin and growth factor signaling pathway. In agreement with this hypothesis, PDK1 protein levels are reduced in the pancreas of E21 rat fetuses [106]. Other genes that are important for the development of pancreatic islet cells, such as *Hnf1a* (hepatocyte nuclear factor-1 alpha), *Hnf4a* (hepatocyte nuclear factor-4 alpha), *Rfx6* (regulatory factor X6), *Pdx1*, *Isl1* (insulin gene enhancer protein), and *Slc2a2* (solute carrier family 2 member 2), show higher expression in islets of 7- and 21-day-old pups from mothers on a LP diet during gestation and lactation [109]. The authors of this study propose that increased expression of these factors is probably an adaptive process to obtain differentiated cells already at birth. This compensatory phenomenon could be very demanding for the cells and could have adverse effects on prematurely differentiated β-cells. In fact, LP neonates have secretory defects and reduced β-cell mass from birth [106,110]. Exposure to an LP diet during fetal life also has effects detectable at the proteomic level. Islets of embryos at E21.5 and cultured 7 days to form pseudo-islets have 45 proteins whose levels are changed in LP islets. The affected proteins include key players in signaling pathways such as energy transduction and redox potentials, glycolysis and Krebs cycle, protein synthesis and metabolism, cell cycle, and differentiation [111]. Although innovative due to the proteomic analysis performed on embryonic islet cells, these results need to be further confirmed in view of the 7-day culture time of these cells isolated from the maternal intrauterine environmental conditions. The same laboratory performed a transcriptomic analysis under the same conditions confirming changes in LP newborn islets in the expression of genes involved in Krebs cycle, electron transport chain and mitochondrial respiration as well as proliferation [112]. Additionally, the LP maternal diet was reported to significantly impact the mTOR pathway in islets of newborns, an effect that persists to adulthood with decreased activity of mTORC1, decreased phosphorylation of ribosomal protein S6 at Ser240 as well as reduced phosphorylation of AKT at Ser473, a known target of mTORC2 [107]. This study also confirmed that microRNAs significantly impact the functional maturation and postnatal development of pancreatic islets in response to an intrauterine LP environment. In fact, the levels of miR-7, miR-152, miR-199a-3p, and miR-342 were increased in the islets of the LP mouse progeny. While miR-7 has previously been shown to regulate the mTOR pathway in adult β-cells [113], miR-199a-3p, and miR-342 are also established regulators mTOR. In fact, lowering the levels of miR-199a-3p and miR-342 restores insulin secretion in the islets of LP offspring at adulthood [107]. Taken together, these data indicate that microRNAs play a key role in nutrient-induced diabetes susceptibility and in the development of metabolic diseases. 

In addition to microRNAs, the long-non-coding RNA H19 seems also to contribute to the dysfunction of β-cells of neonates exposed to maternal malnutrition. H19 levels are inversely correlated with the proliferation capacity of β-cells during postnatal development (see above). H19 expression is decreased earlier in islets of 10-day old LP rats and its decrease may contribute to the reduction of β-cell expansion observed in these animals [18].

Another mechanism proposed to be involved in insulin secretion impairment of LP F1 islets is the dysregulation of the muscarinic acetylcholine receptors subtypes M3 and M2 [114].

Finally, the epigenetic landscape seems to be strongly altered in islets of animals subjected to nutritional deficiency during fetal life (IUGR model) since about 1400 loci showing alterations in cytosine methylation have been detected in the islets of male rats at 7 weeks of age. Multiple genes displaying changes in DNA methylation play relevant roles in β-cell activities and their dysregulation is associated with T2DM development [115]. Differential epigenetic marks on *Pdx1* promoter leads to decreased *Pdx1* expression and correlates with a risk of developing T2DM in adulthood [116]. Another epigenetic phenomenon has been shown to be dysregulated in islets of 3-month-old rats exposed to an LP diet during fetal and early postnatal life [117]. Maternal undernutrition leads to modifications in DNA methylation and histone marks on the hepatocyte nuclear factor 4-α (*Hnf-4a*) locus, a gene required for β-cell differentiation and glucose metabolism [118,119]. These epigenetic marks modify the interaction between the distal P2 promoter and the enhancer region of *Hnf-4a*, leading to a decrease in the expression of the transcription factor. Epigenetic silencing of the Hnf4a locus is accentuated by aging in islets of LP animals compared to control animals [117]. These last studies provide evidence of a link between maternal malnutrition, intrauterine environment, and epigenetic changes in offspring potentially contributing to the susceptibility to develop metabolic disorders. The presence of epigenetic alterations that result from inappropriate in utero conditions, seems to be a phenomenon that increases during aging. Epigenetic alterations resulting from inappropriate in utero conditions are persistent and could even be potentially initiating the deregulations observed with age. In fact, alterations in antioxidant enzyme activity in islets of 3-month-old rats exposed to maternal malnutrition have been observed, reflecting the variations in islet antioxidant potential occurring with age [102]. Again, the physiological response differs between males and females following exposure to an LP diet during fetal and postnatal life, as already observed upon exposure to an “obesogenic” environment. In adulthood, LP males appear more likely than females to develop severe metabolic disorders [110,120]. This is particularly due to an exacerbated mitochondrial dysfunction in LP males, including increased production of reactive oxygen species as well as dysregulation of metabolic genes such as an overexpression of the uncoupling protein-2 [121].

## 5. Conclusions

The postnatal period is a critical stage for the proper development of an organism, especially in the perspective of acquiring cells and organs capable of adapting and compensating for any physio-pathological challenge that may arise in adulthood. Exposure to unfavorable conditions during embryonic, fetal, and neonatal life can have immediate repercussions on health but can also have long lasting effects that persist or are revealed only later in life. An essential point that remains to be further investigated is the impact on β-cell maturation of a deleterious environment restricted either to the prenatal period or to the postnatal period. More studies are needed especially in a context where there is no breastfeeding, since less than 60% of infants are breastfed up to 6 months in the Western world (WHO). Nevertheless, it is currently recognized that a maternal diet during pregnancy and lactation deficient in protein or a diet with an excess of calories and/or lipids leads to replication defects in neonatal β-cells associated with a reduced capacity later in life to adapt the secretory response to metabolic challenges in the case of insulin resistance, obesity, pregnancy, or aging. Thus, the offspring of mothers displaying an inappropriate diet is more likely to display an impaired glycemic control and to develop diabetes, a phenotypic trait that may even be transmitted to future generations. The incidence of metabolic diseases is skyrocketing as a result of the modern lifestyle, which is characterized by a lack of physical activity, increased stress, and the adoption of a Western-style diet that is often too rich in calories and fats. Exposure of fetuses to these deleterious metabolic conditions has a long-term impact on the health of the offspring and should be prevented. We do not yet have a comprehensive picture of the molecular and cellular pathways triggered in β-cells by the exposure to inappropriate nutritional conditions during fetal and postnatal life responsible for the increased susceptibility to diabetes. However, the advent of new technologies allowing global and unbiased analysis of the molecular events taking place in these adverse metabolic conditions promises to shed new light on these processes. A better understanding of the transcriptomic and epigenetic signature of β-cells in the offspring of mothers subjected to malnutrition during pregnancy and lactation may allow the identification of new factors needed for the acquisition of an appropriate number of mature and fully functional β-cells. This will be instrumental for the design of therapeutic strategies to prevent, delay, and treat chronic metabolic diseases including diabetes. The strategy could involve the nutritional education of pregnant women or the development of new pharmaceutical principles. Moreover, this knowledge will help with optimizing experimental approaches to generate fully functional insulin-secreting cells for the replacement of β-cells in Type 1 diabetic patients. 

## Figures and Tables

**Table 1:** The consequences of maternal malnutrition on the progeny metabolism in humans. * Intrauterine growth restriction (IUGR) is a pathological condition defined by an alteration in the expected growth trajectory of the fetus, characterized by low birth weight and impaired organ function.

Maternal Influence	Clinical Signs of the Progeny at Birth and during Childhood	Clinical Signs of the Progeny during Adulthood	References
Famine during pregnancy	➢ Thinness and low weight at birth	➢ Increased body mass index and increased risk for obesity ➢ Reduced lean body mass ➢ Metabolic syndrome ➢ Increased risk for insulin resistance ➢ Glucose intolerance ➢ High prevalence of T2D ➢ Early onset of coronary heart disease	[57,58,59,60]
Pathological pregnancy with intrauterine growth restriction *	➢ Placental epigenetic marks of PDX1 target genes involved in glucose-dependent regulation of the insulin gene expression; correlation of altered epigenetic events with reduced body weight of the newborns ➢ Reduced lean mass and increased percentage body fat up to 2 years of age ➢ Insulin resistance ➢ Reduced transplacental transport of amino acids ➢ Impaired pancreatic islet function and systemic insulin release ➢ Reduced fetal endocrine pancreatic tissue and insulin producing β-cells ➢ Hypoinsulinemia	➢ Persistently reduced lean body mass ➢ Glucose intolerance	[60,61,62,63,64,65,66]
Maternal obesity	➢ Increased body weight at birth and at puberty ➢ Increased incidence of overweight and obesity ➢ Increased insulin resistance at up to 20 years of age ➢ Fetal hyperinsulinemia	➢ Increased body weight ➢ Increased incidence of overweight and obesity ➢ Lower circulating high density lipoproteins ➢ 29% increased risk of death caused by cardiovascular disease	[67,68,69,70,71,72,73,74]
Gestation diabetes mellitus	➢ Macrosomia ➢ Hypoglycemia ➢ Infancy obesity	➢ Metabolic syndrome ➢ Excess abdominal adiposity ➢ Hyperinsulinemia ➢ Dysglycemia ➢ Obesity ➢ T2D ➢ Cardiova6scular disease	[75,76]

## Abbreviations

ABCC8	ATP binding cassette subfamily C member 8
ADP	adenosine diphosphate
AKT	a serine/threonine protein kinase
AMPK	AMP-activated protein kinase
ATP	adenosine triphosphate
Atp5f1	ATP synthase peripheral stalk-membrane subunit B
BMP4	bone morphogenetic protein 4
Ca^2+^	calcium ions
Cacna1c/d	calcium voltage-gated channel subunit alpha1 C/D
CASR	calcium-sensing receptor
CCL2	C-C Motif Chemokine Ligand 2
Ccnc	cyclin C
Ccnd1/2	cyclin D1/2
CDK1	cyclin-dependent kinase 1
Cdt1	chromatin licensing and DNA replication factor 1
CENPA	centromere protein A
c-Myc	myelocytomatosis viral proto-oncogene, BHLH transcription factor
Deptor	DEP domain containing MTOR interacting protein
Dlk	dual leucine zipper-bearing kinase
DNA	deoxyribonucleic acid
DNMT3A	DNA methyltransferase 3 alpha
ETC	electron transport chain
Fabp5	fatty acid-binding protein 5
FACS	fluorescence-activated cell sorting
Fev	fifth ewing variant
Fltp	flattop
FOXM1	forkhead box protein M1
GAPDH	glyceraldehyde-3-phosphate dehydrogenase
Gas6	growth arrest specific protein 6
Gck	glucokinase
Glp1	glucagon-like peptide 1
GSIS	glucose-stimulated insulin secretion
H19	H19 imprinted maternally expressed transcript
H3K4	histone H3 lysine K4
HFD	high-fat diet
Hk	hexokinase 1
Hnf1a/4a	hepatocyte nuclear factor-1/4 alpha
IL1beta	interleukin 1 beta
IL-1Ra	interleukin 1 receptor antagonist
Il13ra2	interleukin 13 receptor subunit alpha 2
iPSC	induced pluripotent stem cells
Isl1	insulin gene enhancer protein
IUGR	intrauterine growth retardation
Jak	janus kinase
Jnk3	c-Jun N-terminal kinase 3
K^+^	a positively charged potassium ion
Ldha	lactate dehydrogenase A
LP	low protein
Maea	macrophage erythroblast attacher, E3 ubiquitin ligase
Mafa	basic leucine zipper (bZIP) transcription factor A
Mafb	basic leucine zipper (bZIP) transcription factor B
Mcm3/4/6/7/10	minichromosome maintenance complex component 3/4/6/7/10
Mct1	monocarboxylate transporter 1
Mettl3/14	RNA methyltransferase-like 3/14
MIN6	mouse insulinoma cell line
mTORC1/2	mammalian target of rapamycin complex 1/2
NADH	nicotinamide adenine dinucleotide
NeuroD1	neuronal differentiation 1
Nkx6.1	NK6 homeobox 1
Orc5/6	origin recognition complex subunit 5/6
OxPhos	oxidative phosphorylation
PCP	planar cell polarity
Pdgfrα	platelet derived growth factor receptor alpha
Pdk1	phosphoinositide-dependent kinase-1
Pdx1	pancreatic and duodenal homeobox 1
PI3K	phosphoinositide 3-kinase
PLA2	phospholipase A2
Prim1	DNA Primase Subunit 1
Prlr	prolactin receptor
PYY	peptide YY
Rest	RE1 silencing transcription factor
Rfx6	regulatory factor X6
RNA	ribonucleic acid
Rps6/14	ribosomal protein S6/14
SIX3	Sine Oculis homeobox homolog 3
Slc2a2	solute carrier family 2 member 2
SMAD2/3	TGFB signaling protein, mothers against decapentaplegic homolog 2/3
Sod2	superoxide dismutase 2
Stat3	signal transducer and activator of transcription 3
T2DM	type 2 diabetes mellitus
TCA	tricarboxylic acid cycle
Tfam	transcription factor A, mitochondrial
TGFBR	transforming growth factor beta receptor
TH	thyroid hormone
TK	transketolase
TOP2A	DNA Topoisomerase II Alpha
tRF	tRNA-derived fragment
tRNA	transfer RNA
Trpm5	transient receptor potential cation channel subfamily M member 5
Ucn3	urocortin 3
Vipr1	vasoactive intestinal polypeptide receptor 1
Wnt	wingless/integrated
